# Light Entrained Rhythmic Gene Expression in the Sea Anemone *Nematostella vectensis*: The Evolution of the Animal Circadian Clock

**DOI:** 10.1371/journal.pone.0012805

**Published:** 2010-09-21

**Authors:** Adam M. Reitzel, Lars Behrendt, Ann M. Tarrant

**Affiliations:** Biology Department, Woods Hole Oceanographic Institution, Woods Hole, Massachusetts, United States of America; Vanderbilt University, United States of America

## Abstract

**Background:**

Circadian rhythms in behavior and physiology are the observable phenotypes from cycles in expression of, interactions between, and degradation of the underlying molecular components. In bilaterian animals, the core molecular components include *Timeless*-*Timeout*, photoreceptive cryptochromes, and several members of the basic-loop-helix-Per-ARNT-Sim (bHLH-PAS) family. While many of core circadian genes are conserved throughout the Bilateria, their specific roles vary among species. Here, we identify and experimentally study the rhythmic gene expression of conserved circadian clock members in a sea anemone in order to characterize this gene network in a member of the phylum Cnidaria and to infer critical components of the clockwork used in the last common ancestor of cnidarians and bilaterians.

**Methodology/Principal Findings:**

We identified homologs of circadian regulatory genes in the sea anemone *Nematostella vectensis*, including a gene most similar to *Timeout*, three cryptochromes, and several key bHLH-PAS transcription factors. We then maintained *N. vectensis* either in complete darkness or in a 12 hour light: 12 hour dark cycle in three different light treatments (blue only, full spectrum, blue-depleted). Gene expression varied in response to light cycle and light treatment, with a particularly strong pattern observed for *NvClock*. The cryptochromes more closely related to the light-sensitive clade of cryptochromes were upregulated in light treatments that included blue wavelengths. With co-immunoprecipitation, we determined that heterodimerization between CLOCK and CYCLE is conserved within *N. vectensis*. Additionally, we identified E-box motifs, DNA sequences recognized by the CLOCK:CYCLE heterodimer, upstream of genes showing rhythmic expression.

**Conclusions/Significance:**

This study reveals conserved molecular and functional components of the circadian clock that were in place at the divergence of the Cnidaria and Bilateria, suggesting the animal circadian clockwork is more ancient than previous data suggest. Characterizing circadian regulation in a cnidarian provides insight into the early origins of animal circadian rhythms and molecular regulation of environmentally cued behaviors.

## Introduction

Most organisms exhibit daily physiological and behavioral rhythms that are regulated by molecular circadian clocks. Although light is the most common signal entraining these rhythms, other environmental signals such as food availability and temperature can also drive oscillations in gene expression through the same molecular clock [1], [2]. Characterization of the genes composing clocks in diverse organisms (e.g., bacteria [3], plants [4], fungi [5], animals [6]) has suggested that the circadian clock has evolved independently many times [7].

In animals, the molecular components of the circadian clock have been exquisitely characterized in mammals and diverse insects [8], [9]. From these studies, it is clear that many of the core clock genes are conserved in these two disparate animal groups [10], suggesting that this molecular clock dates back to at least the ancestor of deuterostomes and protostomes. Both mammalian and insect clocks are based on two interacting molecular feedback loops: a positive loop driven by expression of the bHLH-PAS transcription factors C*lock* and C*ycle* (*Bmal1*/*Mop3* in mammals) that then heterodimerize to regulate downstream gene expression through E-box motifs, and a negative loop where dimerization of PERIOD and cryptochrome paralogs (mammals) or PERIOD and TIMELESS (*Drosophila*) inhibit the CLOCK:CYCLE heterodimer [10]. Despite broad similarities, studies of additional insect species have revealed a number of variations of the negative loop involving cryptochromes and *Timeless* [11], [12]. The divergence of molecular components and their function between mammals and insects and within insects can be explained, in part, by gene duplication and loss [12], [13].

Several studies comparing gene families between bilaterians and cnidarians have suggested that their most recent common ancestor had a complex genome [14], [15]. Most of these studies have focused on transcription factors involved in embryogenesis and morphogenesis, leaving unknown to what extent the molecular components that regulate physiology and behavior are conserved. Because the core genes that regulate circadian cycling are largely conserved within the Bilateria, we hypothesized that the circadian machinery would operate similarly in cnidarians. Understanding the mechanics of the cnidarian clock will reveal which genes likely composed the clock of the ancestor to the Cnidaria and Bilateria, which was certainly a marine species living in an environment dominated by blue light [16].

Cnidarian physiology and behavior strongly vary on diel cycles. In some cnidarians, skeletal growth and energetic metabolism are strongly coupled with photosynthesis by algal endosymbionts [17]. However, other behaviors, such as spawning, tentacle extension and feeding, may also be dependent on regular cycling of light:dark periods and variations in subjective day length [18], [19]. In the only study of circadian-like gene expression in a cnidarian, Levy et al. [20] identified two cryptochromes from the coral *Acropora millepora* that displayed rhythmic gene expression in response to a light:dark treatment. Notably, one of these cryptochromes was upregulated during full moon, prior to spawning. Indeed, corals are extremely sensitive to low levels of blue light, consistent with detection of lunar irradiation [21]. Together, the behavioral and molecular studies suggest that cnidarians may share conserved molecular elements in the circadian clock with insects and mammals.

In this study, we have identified key components of the circadian clock in a model cnidarian, the starlet sea anemone, *Nematostella vectensis.* For this work, *N. vectensis* has several advantages including a sequenced genome, ease of laboratory culture, a simple, transparent body column, and absence of algal symbionts, which allows for more direct interpretation of cnidarian-specific responses [14], [18], [22]. Previous research has shown that gametogenesis by *N. vectensis* can be strongly influenced by light:dark cycling [23] and that *N. vectensis* contains likely homologs to the bilaterian clockwork [24]. Through bioinformatic searches and phylogenetic analyses we identified conserved circadian genes from *N. vectensis*. We then quantified the expression of circadian genes in response to light:dark cycles as well as in response to different portions of the light spectrum. We also tested for conserved protein-protein interactions between *N*. *vectensis*' orthologs to *Clock* and *Cycle* and identified canonical E-box motifs in the promoters of *NvClock* and one cryptochrome, two genes that showed rhythmic gene expression. Together, our data show that two components of the animal circadian clock, cyclic expression of orthologous circadian genes and heterodimerization of key bHLH-PAS proteins, were in place at the divergence of the Cnidaria and Bilateria.

## Results and Discussion

**Figure 1:**
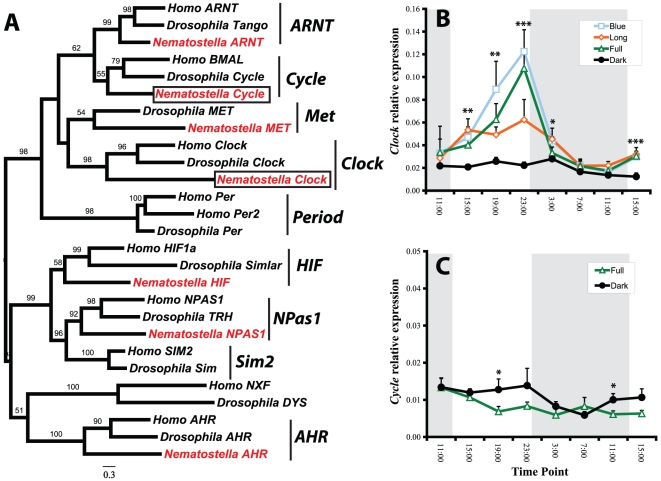
Phylogenetic identification and light-dependent, rhythmic expression of bHLH-PAS genes in *Nematostella*. (A) Maximum likelihood analysis of bHLH-PAS genes from *Nematostella vectensis*, *Homo sapiens*, and *Drosophila melanogaster*. Anemone orthologs to *Clock* (XP_001639742) and *Cycle* (XP_001624731) (boxed), as well as other bHLH-PAS genes are shown in red. Numbers above nodes indicate percentage of 1000 bootstraps. Bootstraps below 40 were removed. Temporal gene expression of (B) *NvClock* under three light treatments and constant dark and (C) *NvCycle* under full spectrum and dark. *NvClock* was significantly upregulated during subjective day and the magnitude of upregulation was dependent on light treatment. *NvCycle* showed similar expression in full spectrum and constant dark treatments with small differences in expression in response to light cycling. When significant differences were observed between treatments, expression of *NvCycle* was higher in constant dark treatments. Asterisks indicate significant difference among treatments (* <0.05, ** <0.001, *** <0.0001) and error bars are + s.d. Post-hoc analysis groupings for *NvClock*: 15:00, (B,L,F)D; 19:00, (B,F)(L,D); 23:00, (B,F)L,D; 3:00, (B,L,F)D; 15:00, (B,L,F)D. Abbreviations: B  =  Blue, F  =  Full, L  =  Long, and D  =  Dark. Alternative use of parentheses and underlining indicates statistically indistinguishable treatments at each time-point.

**Figure 2:**
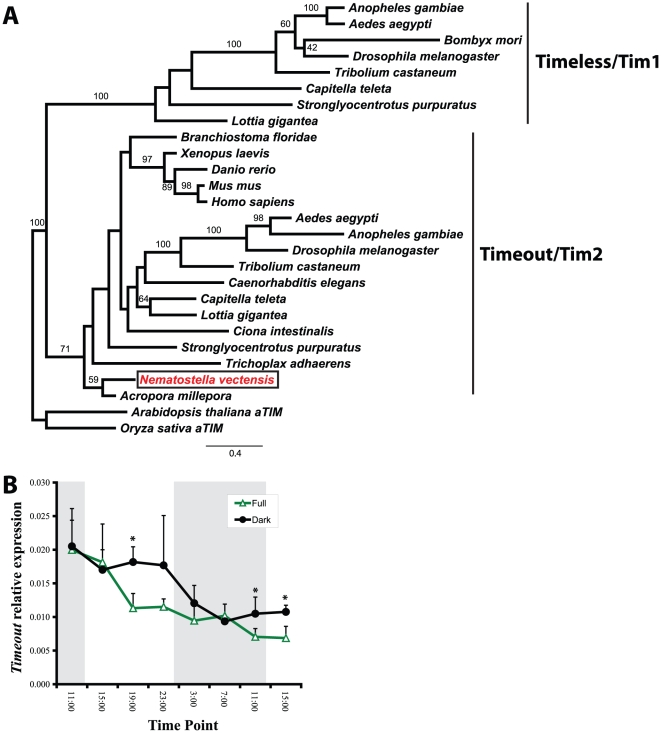
Phylogenetic identification and light-dependent, rhythmic expression of *Timeout* in *Nematostella*. (A) Identification of a single gene from *N. vectensis*) (XP_001641000) most similar to *Timeout*/*tim2* (boxed, red) through maximum likelihood analysis. *Timeout* and *Timeless* cluster as two independent clades resulting from a gene duplication prior to the bilaterian ancestor. We identified supported *Timeless*/*tim1* genes from two lophotrochozoans, a mollusc and an annelid, that group with orthologs from insects and sea urchin previously reported by Rubin *et al*. [30]. *NvTimeout* groups with high bootstrap support with a *Timeout* gene from the coral *A. millepora* (GenBank Accession: EZ013923). (B) Temporal gene expression of *NvTimeout* under full light treatment and constant darkness showed little transcription differences among full spectrum and constant dark, but a significant decline in expression over the course of the experiment in both treatments (p<0.0001). The observed significant differences (* <0.05) between treatments that had higher expression in the constant dark treatment were not consistent with a time in the light cycle, and represented small differences in gene expression. Error bars are + s.d.

**Figure 3:**
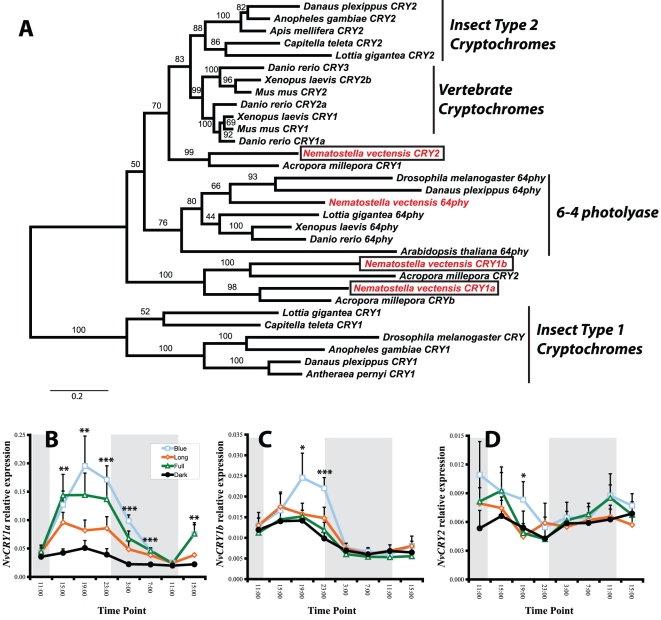
Phylogenetic identification and light-dependent, rhythmic expression of cryptochromes in *Nematostella*. (A) Phylogenetic relationships of three *N. vectensis* cryptochromes (“CRY,” red, boxed, 2: XP_001623146, 1a: XP_001631029, 1b: XP_001632849), as well as an ortholog to 6-4 photolyase (“64phy,” red, XP_001636303) and cryptochromes from diverse protostomes and deuterostomes. All three *N. vectensis* cryptochromes group with high support with cryptochromes from the coral *A. millepora* (two of these reported in [20], one unreported (*CRYb*, GenBank Accession: DY585180)). We have named the *N. vectensis* cryptochromes in a manner consistent with the nomenclature used for bilaterian genes and not the previously published coral nomenclature, which did not reflect relationships to recognized gene families. The cnidarian cryptochromes belong to two clades: one clade forms an outgroup to the vertebrate plus insect Type 2 cryptochromes, and the second is a cnidarian-specific duplication that groups between Type 1 cryptochromes and 6-4 photolyase from diverse species. Additionally, we identified insect Type 1 and 2 cryptochromes in two lophotrochozoans (*Lottia gigantea* and *Capitella teleta*). The tree was mid-point rooted. (B-D) Temporal gene expression of *NvCry1a*, *1b*, and *2* from three light treatments and constant dark show a diverse degree of transcriptional regulation. (B) *NvCry1a* was significantly upregulated in subjective day in all light treatments with higher mean expression in adults in the full-spectrum and blue-light treatments. When light was removed, expression decreased in all treatments but remained significant throughout more than half of subjective night. (C) *NvCry1b* was only upregulated in the blue LED light treatment during the later portion of subjective day. (D) *NvCry2* showed no differences in expression over the course of the experiment in any treatment except at one time point where expression was highest in the blue LED light treatment. Although statistically significant (p = 0.01), the mean expression represented only a 0.5-fold increase in transcription when compared with the other light treatments. Asterisks indicate significant difference among treatments (* <0.05, ** <0.001, *** <0.0001) and error bars are + s.d. Post-hoc analysis groupings for *NvCry1a*: 15:00, (B,L,F)D; 19:00, (B,F)(L,D); 23:00, (B,F)(L,D); 3:00, B(L,F)D; 7:00, (B,L,F)D, 15:00, (B,F)(L,D). Post-hoc analysis groupings for *NvCry1b*: 19:00, B(F,L,D); 23:00, B(F,L)D. Post-hoc analysis groupings for *NvCry2*: 19:00, (B,D)F,L. See Figure 1 legend for description of abbreviations and designation of statistical groups.

Through bioinformatic queries and phylogenetic analyses we identified a number of *Nematostella vectensis* orthologs to core circadian clock genes shared by vertebrates and insects. From the bHLH-PAS family, *N. vectensis* has strongly supported orthologs to *Clock* and *Cycle* [as previously reported in 25], as well as a number of other bHLH-PAS transcription factors (Figure 1A). There are no orthologs to *Period* in *N. vectensis* or the coral *A. millepora*, despite an earlier report suggesting these two cnidarians may have these genes [[24], see Figure S1]. Indeed, *Period* genes have not been identified in any non-bilaterian phylum [25], suggesting this subfamily of transcription factors evolved within the Bilateria. We identified a single gene with high similarity to *Timeout* from human and *Timeless*/*Timeout* from diverse insects (Figure 2a). We also identified three cryptochromes, two of which are orthologs to previously reported genes from the coral *A. millepora* (Figure 3a, [20]). Additionally, we previously showed that *N. vectensis* lacks orthologs to the nuclear receptors *Rev-Erb* (NR1D1) and *ROR* (NR1F1) [26], important components of the mammalian clock.

*Clock* and *Cycle* proteins form the core of the positive regulatory loop of the circadian clock in both mammals and insects. However, the expression of these transcription factors during a light cycle differs between these taxa [reviewed in 10]. *NvClock* showed strong diurnal expression with peak expression in the mid- to late subjective day (Figure 1B, zeitgeber time (ZT)  = 6–12). The magnitude and timing of peak expression were significantly influenced by the light quality, with higher expression and a slightly shifted peak in the two light treatments with a high proportion of blue wavelengths. *NvCycle* expression showed small differences between the full spectrum and dark treatments over the sampled time points (Figure 1C). Together, the pattern of rhythmic expression for these two bHLH-PAS transcripts was more similar to *Drosophila*, where *DmClock* exhibits peak expression in the light portion of a light:dark cycle, albeit at an earlier portion of this period (ZT  = 0–4) [27], [28]. In contrast, *Clock* expression in mouse does not vary during a light:dark rhythm [29].

**Figure 4:**
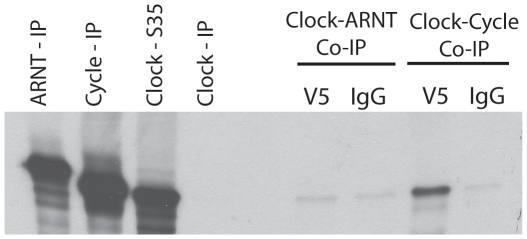
Heterodimerization of *Nematostella* CLOCK and CYCLE. Co-immunoprecipation showing evidence for specific dimerization of NvCLOCK and NvCYCLE. Full length constructs of NvCLOCK were synthesized with [35S]methionine, incubated with unlabeled NvCYCLE and NvARNT, and then co-immunoprecipated using an antibody to V5-epitope (V5) or normal mouse IgG (IgG). NvCLOCK did not dimerize with NvARNT, the most closely related bHLH-PAS protein to CYCLE (see Figure 1). The first two lanes from left (Lanes 1 and 2) show control reactions of NvCYCLE and NvARNT synthesized with [35S]methionine and immunoprecipated with the antibody to the V5-epitope.

A shared protein-level interaction of mammalian and insect circadian clocks is the heterodimerization of CLOCK and CYCLE that together act as transcriptional regulators. In mammals, the CLOCK:CYCLE heterodimer upregulates expression of *Period* paralogs, among other genes. In *Drosophila*, as well as some other insects, the CLOCK:CYCLE dimer upregulates *Period* and *Timeless*, as well as other transcriptional targets. For *N. vectensis*, co-immunoprecipitation of NvCYCLE and NvCLOCK revealed that these two genes specifically dimerize *in vitro* (Figure 4). No non-specific dimerization was observed between NvCLOCK and NvARNT, a bHLH-PAS transcription factor closely related to *Cycle* (see Figure 1A). These data support the hypothesis that the dimerization of CLOCK and CYCLE dates back to at least the cnidarian-bilaterian ancestor and is likely an ancient component of the animal circadian clock.

The Timeless-Timeout family contains two paralogs in *Drosophila* and other insects but only one gene in diverse vertebrates, invertebrates, and non-animal species [30], [31]. Members of the Timeless-Timeout family in vertebrates are orthologous to *Timeout* from insects but have been frequently referred to as “*Timeless*”, likely due to their description prior to the identification of the paralogs in *Drosophila*. Early descriptions of the taxonomic distribution of genes in the Timeless-Timeout family suggested an insect-specific gene duplication [31]. However, Rubin et al. [30] identified both *Timeless* and *Timeout* in the sea urchin *Stronglyocentrotus purpuratus* indicating that this gene duplication event occurred prior to the divergence of protostomes and deuterostomes. We recovered *Timeless* and *Timeout* orthologs from additional protostomes (Figure 2A), supporting a hypothesis these paralogs are present broadly in bilaterian animals, with *Timeless* independently lost in diverse lineages (e.g., chordates, nematodes). We recovered only one gene from each surveyed cnidarian and placozoan, further supporting a conclusion that the gene duplication event occurred after the cnidarian-bilaterian divergence but before the protostome-deuterostome split. The genes from these early diverging phyla, including the gene from *N. vectensis*, shared greater sequence similarity to *Timeout* and grouped with these genes in the phylogenetic analysis (Figure 2A).

*NvTimeout* expression did not vary consistently in response to light treatment (Figure 2B). The role of *Timeless* in circadian gene regulation was originally reported in *Drosophila*, where expression follows a 24-hr cycle [32] through interaction with the CLOCK:CYCLE heterodimers [28], [32]. *Timeless* is part of the circadian clock for some other insects besides *Drosophila* [33], [34], but other insects lack *Timeless* completely [30]. In addition, *Timeout* has recently been shown to play dual roles in *Drosophila* as part of the circadian clock and in chromosome stability [35]. Despite conflicting data showing an inconclusive role for *Timeout* in the mammalian circadian clock [36], recent work has provided evidence that *Timeout* indeed plays a critical role in regulating the mouse clock [37]. Similar to *Drosophila*, mammalian *Timeout* has a second function independent of the circadian clock as one component of the DNA replication fork complex [38]. Because *Timeout* and its binding partner *Tipin* are well conserved in both animals and fungi, the ancestral role of these proteins may have been in regulation of DNA replication rather than circadian cycling [38].

The role, if any, for *Timeout* from *N. vectensis* in the circadian clock is presently uncertain. *NvTimeout*, like vertebrate and insect *Timeout* genes, has poorly conserved first *Period*-interacting and cytoplasmic localization domains, and lacks a conserved nuclear localization signal (Figure S2). In addition, *NvTimeout* lacks the DEDD portion of the cytoplasmic localization domain, which is conserved in vertebrates and insects. Through bioinformatic searches, we identified a homolog of *Tipin* from *N. vectensis* (XP_001625500), suggesting that NvTIMEOUT may interact with TIPIN to regulate DNA replication. In addition, because *N. vectensis* and other early diverging animals lack *Period* genes, we have no hypothesis for a binding partner for TIMEOUT in a potential repressor loop of the circadian clock. Future studies testing whether NvTIMEOUT is degraded in a light-dependent manner, as shown in *Drosophila* [39], would be particularly informative.

We identified three cryptochromes from *N. vectensis*. Two are supported as a cnidarian-specific duplication of an ancestral gene that groups between Type 1 cryptochromes and the DNA repair enzyme 6-4 photolyase, and the third groups with vertebrate and insect Type 2 cryptochromes (Figure 3A). In *N. vectensis*, expression of these three cryptochromes varied in response to the light treatments and time in the light cycle. *NvCry1a* expression significantly increased in response to all three light treatments with the highest expression in those treatments with blue wavelengths (Figure 3B, *i.e*., full spectrum and blue-only). Transcription increased immediately after the light period started, was sustained while light was present, and decreased rapidly once light was removed. Anemones cultured in continuous darkness showed no cycling, suggesting that the response was a result of exposure to light, not an unmeasured variable or an endogenous signal. *NvCry1b*, an ortholog of *AmCry2* previously reported from the coral *A. millepora*, had significant diel cycling only in the blue-light only treatment (Figure 3C). Transcription increased midway through the light period and then decreased once light was removed. In *A. millepora*, this cryptochrome showed a similar rhythmic transcriptional response. Interestingly, *AmCry2* was shown to be responsive to moonlight, which is particularly enriched with blue light. Taken together, these data suggest that this cnidarian-specific cryptochrome may have evolved sensitivity to a narrow band of the light spectrum early in the anthozoan lineage.

The third *N. vectensis* cryptochrome, *NvCry2*, showed no strong response to diurnal light cycling for any light treatment (Figure 3D). These results were of interest for two reasons. First, *NvCry2* expression in a light:dark cycle was strikingly different from that of the closely related cryptochrome *AmCry1* from *A. millepora*. The coral cryptochrome had strong diurnal expression that peaked during the daylight, similar to *AmCry2*. Thus, the coral and anemone orthologs, despite similar evolutionary history, have strongly divergent expression in response to light treatment. Secondly, *NvCry2* and *AmCry1* are most similar to previously characterized vertebrate and insect Type 2 cryptochromes. Vertebrate and insect Type 2 cryptochromes are derived from 6-4 photolyase and act as repressors in insects and mammals [12], [34], [40], [41]. In mammals, cryptochrome expression is offset from *Cycle* [41]. Unlike the mammalian pattern, *NvCry2* expression was not offset from the putative positive regulators *NvCycle* and *NvClock*. *NvCry2* expression was more similar to the pattern observed in some insects (*e.g*., monarch and mosquito), in which expression of these insect *Cry2* genes does not significantly vary over a light:dark period [12], [42]. However, these insect proteins still act as a critical part of the negative circadian regulatory loop. It is plausible that *NvCry2* could similarly act as a repressor (discussed below).

We searched *N. vectensis* promoter sequences for evidence of conserved motifs known to regulate circadian signaling. In mammals and insects, CLOCK:CYCLE heterodimers regulate transcription of downstream genes by binding to E-box motifs (CACGTG) [43], [44]. We searched for E-box motifs in the 2 kb upstream of the transcriptional start-sites of the six putative circadian genes we have identified in this study. We identified identical matches near the start site in promoters for two genes: one for *NvClock* (-225 bp) and two for *NvCry1a* (−228, −526 bp). These genes were the most strongly upregulated during subjective day. The only other gene in our survey that displayed rhythmic expression was *NvCry1b*, which has an E-box motif ∼1800 bp upstream of the start site. These data provide correlative evidence that the *N. vectensis* CLOCK:CYCLE heterodimer may regulate transcription of target genes through the same DNA motifs that have been identified in mammals and insects. Currently, there have been few studies assessing the conservation of bilaterian regulatory motifs in early diverging animals. One study has shown conserved specificity for DNA binding motifs for the transcription factor NF-κB from *N. vectensis* when compared with other animals [45]. Thus, it would not be unexpected that NvCLOCK:CYCLE heterodimers may regulate transcription of downstream genes through canonical E-box motifs.

One likely divergence in the *N. vectensis* clock from other animals is the composition of the negative regulatory loop. *N. vectensis* lacks *Period* genes, and *NvTimout* expression did not vary in response to a daily light cycle. The negative regulatory loop differs among mammals and insects, partially as a result of gene duplication and loss within each lineage (duplication of *Period* in mammals, loss of *Timeless* and CRY1 and 2 cryptochromes in some insects, see [12], [13]), so a novel loop in cnidarians may not be surprising. As discussed above, one likely component of the negative regulatory loop in *N. vectensis* is *NvCry2*. Cryptochromes play a critical portion of the negative regulatory loop in both vertebrates [40], [41] and nondrosophilid insects [12], [34], [42] by inhibiting CLOCK:CYCLE mediated transcription. Given that *N. vectensis* lacks *Period* genes, it is plausible that *NvCry2* independently inhibits the positive loop, as has been shown in these other animals. The transcription of *NvCry2* did not correlate with light exposure, similar to mCRY2 from mouse [41] and CRY2 from diverse insects [12], suggesting that this gene does not function as a photoreceptor. Future research studying whether *NvCry2* protein suppresses CLOCK:CYCLE –mediated transcription would be particularly informative for understanding the potential role of this protein in the negative loop of the cnidarian circadian clock. Additionally, a repressive role by this anemone protein would further push back the date for the function of Type 2 cryptochromes in the animal circadian clock.

Our combined study of gene representation, rhythmic gene expression, and conserved protein-protein interactions suggest that the circadian clock from *N. vectensis* is similar to those reported from mammals and insects, and thus many molecular aspects of the circadian oscillator were present in the cnidarian-bilaterian ancestor. In addition, some molecular components of the circadian clock may be even more ancient. Orthologs to *Clock* and a presumptive *ARNT*/*Cycle* ancestral gene [25] as well as a cryptochrome [46] have been reported from sponges. Sponge behavior is also responsive to diel light cycles [e.g., 47]. Future work with sponges and other early diverging animals, including characterization of protein-protein interactions and DNA regulatory motifs, as we have reported here for *N*. *vectensis*, will further elucidate which components of the animal circadian clock were likely present at the origin of the animal kingdom.

## Materials and Methods

### Gene identification and classification

*Nematostella vectensis* representatives of the Timeless-Timeout family, cryptochromes, and several members of the bHLH-PAS family were identified through BLASTp searches of protein models through the JGI browser. Gene models were modified through EST searches at JGI and NCBI. We used a likelihood-based approach to determine evolutionary relationships of the *N. vectensis* genes with a combination of outgroup sequences appropriate for each gene family. For the bHLH-PAS family, we used sequences from *Homo sapiens* and *Drosophila melanogaster* to represent the diversity of subfamilies. For the cryptochrome analysis, we used sequences from various insects and vertebrates and three coral cryptochromes (sequences from [12], [20], [42] plus selected additional sequences (*Lottia gigantea* (*LgCry1*: JGI: 143285, *LgCry2*: 131547), *Capitella teleta* (*CtCry1*: JGI:226189, *CtCry2*: 178510)). For phylogenetic analyses of *Timeless*/*Timeout*, we used sequences from diverse insects and various other animals (sequences from [30] plus (*Lottia gigantea* (TO: JGI: 206053; Tim: 170270), *Capitella teleta* (TO: JGI: 19701; Tim: 202856), *Trichoplax adhaerens* (GenBank: XP_002110029)) and aTIM from two plants (*Arabidopsis thaliana*, NP_200103; *Oryza sativa*, NP_001054915), which have high sequence similarity to *Timeless*/*Timeout*. Full length sequences for all taxa were aligned with Muscle 3.6 [48] and edited manually in the case of clear errors and to remove gaps. Maximum likelihood analyses were run using RAxML (version 7.0.4) [49] with the optimized protein models (model determined by AIC criteria with ProtTest, [50]). Trees were visualized and illustrated with FigTree v1.1.2 (http://tree.bio.ed.ac.uk/software/Figtree/).

### Animal culture and experimental treatments

Adult *N. vectensis* were maintained at 25°C as previously described [26] in six separate glass dishes per treatment. Experimental light treatments were either all dark or a 12∶12 light:dark treatment. The three light treatments (“full spectrum”, Corallife 50/50 bulb; “blue light”, blue LED; “long wavelength”, fluorescent bulb relatively depleted in blue wavelengths) represented different portions of the light spectra, as measured by an Ocean Optics USB4000 spectrometer (see Figure S3 for light spectra). Individuals were cultured in their respective light treatment for one month prior to sampling; culturing conditions were otherwise identical for each group. Samples were collected every four hours over a 28-hr time period. At each time, four replicate samples of 8-10 individuals were removed haphazardly from each treatment, and immediately preserved in RNAlater and stored at -20°C.

### cDNA synthesis and quantitative PCR

Anemone tissues were mechanically homogenized; RNA was purified, DNAse treated, and quantified using a nanodrop spectrophotometer, as previously described [26]. RNA integrity for selected samples was checked with denaturing gel electrophoresis. cDNA was synthesized with the iScript cDNA Synthesis Kit (Bio-Rad) using 1.5 µg of total RNA in a 30 µl reaction. For each gene of interest, we produced a plasmid standard from an amplified portion of each transcript cloned into pGEMT-easy (Promega). The standard curve was used in qPCR reactions to quantify amplification efficiency and to calculate the number of molecules per reaction [as in 26]. qPCR primers were designed, as previously described [[26], see Table S1 for primers]. qPCR was performed with a MyCycler Real-Time PCR detection system using iQ SYBR Green Supermix (Bio-Rad). For each gene, standards were run in duplicate wells and experimental samples were run on a single plate. The PCR mixture consisted of 11.5 µl of molecular biology grade distilled water, 12.5 µl of IQ SYBR Green Supermix, 0.5 µl of 10 µM gene-specific primers, and 0.5 µl of cDNA. PCR conditions were as follows: 95°C for 3 min; 40 cycles of 95°C for 15 s and 64°C for 45 s. After 40 cycles, the PCR products from each reaction were subjected to melt curve analysis to ensure that only a single product was amplified. The number of molecules per µl for each gene was calculated by comparing the threshold cycle (Ct) from the sample with the standard curve. Expression for each gene was normalized to a constitutive heat shock protein (HSC71) from *N. vectensis*, which had little change in expression among treatments or time points. Expression was compared among treatments using ANOVA with Tukey's Honestly Significant Difference Test as a posthoc test.

### Co-immunoprecipitation

Full-length *NvCycle*, *NvClock*, and *NvARNT* were amplified with cDNA synthesized from polyA-RNA (see Table S2 for primers, Figures S4, S5 and S6 for full sequences and translation). *NvCycle* and *NvARNT* products lacked the stop codon to facilitate addition of the V5-epitope to the C-terminus. The *NvClock* PCR product included the endogenous stop codon. All PCR products were cloned in the Gateway vector pENTR (Invitrogen) and transformed into Top10 cells. Cloned sequences were confirmed by comparison with predicted transcripts from gene annotation. These products were then transformed into pcDNA 3.2-DEST (Invitrogen). Co-immunoprecipitation was performed as described by Evans et al. [51]. *NvCycle*, *NvClock*, and *NvARNT* proteins were synthesized by *in vitro* transcription and translation (TnT, Promega) in the presence of [^35^S]methionine. NvCYCLE and NvARNT were additionally synthesized in the absence of radioactivity for co-immunoprecipitation. For co-immunoprecipitation reactions, 5 µl of unlabeled protein was mixed with 15 µl of radiolabeled protein and incubated at room temperature (RT) for 2 hours. For control IP reactions, 5 µl of labeled protein was used. All protein reactions were adjusted to 100 µl with 1.25X IP buffer and precleared in two steps (1 hour at RT, overnight at 4°C) with mouse IgG and protein agarose G. Five µg of monoclonal anti-V5 antibody (Invitrogen) or mouse IgG was added to the appropriate reactions and incubated at RT for 2 hours. Products were precipitated overnight at 4°C with protein agarose G. The agarose beads were washed twice with 1X IP buffer, boiled in sample treatment buffer, and run on SDS-polyacrylamide gel electrophoresis on an 8% gel. Gels were visualized by fluorography on photographic paper after overnight exposure.

### Promoter searches for E-box motifs

Two kilobases upstream of each start site for each gene was searched for canonical E-box motifs (CACGTG). Where identified, the site for each motif was annotated based on position upstream of the start-site.

## Supporting Information

Figure S1 Identity of genes determined by phylogenetics previously annotated as cnidarian Period representatives by Vize [52]. In this earlier study, top BLAST matches of cnidarian gene models and ESTs to mammalian and insect Period were reported. In our analysis, we show that none of these (indicated by arrowheads in figure) are orthologs to Period genes from mammals or insects, which form a strongly supported clade (represented by DmPer, HsPer, and HsPer2). The three *Acropora* genes (Am) group with genes from *Nematostella* (Nv) that nest within three separate bHLH-PAS families: HIF, ARNT, and Bmal. Phylogenetic methods are identical to those used throughout this study. Support values above nodes indicated percentage of 1000 bootstraps.
(0.04 MB DOCX)

Figure S2 Alignment of *Timeless-Timeout* genes from vertebrates, insects, and *N. vectensis*. We have focused on three functional domains: Period interacting domain 1 that contains the nuclear localization signal, Period interacting domain 2, and the cytoplasmic interaction domain [defined in 53]. The single *N. vectensis* gene with similarity to the *Timeless-Timeout* family has considerably higher conservation with vertebrate *Timeout* and insect *Timeout* for two of the three domains than insect *Timeless*. As a representative example, the percent conservation of *NvTimeout* is higher when compared with human *Timeout* and *Drosophila Timeout* than with *Drosophila Timeless* (Period domain 1∶28, 32, 7%, respectively; Period Domain 2∶42, 29, 12%, respectively). The cytoplasmic localization domain could not be aligned with confidence outside of the DEDD region (which *N. vectensis* lacks) for these taxa and thus percent similarities could not be calculated.
(0.29 MB DOCX)

Figure S3 Light spectra from the four experimental treatments used in this study. Spectra were determined with a USB4000 spectrometer (Ocean Optics, Dunedin, FL).
(0.06 MB DOCX)

Figure S4 Assembled transcript and open reading frame for *Nematostella Clock*.
(0.03 MB DOC)

Figure S5 Assembled transcript and open reading frame for *Nematostella Cycle*.
(0.03 MB DOC)

Figure S6 Assembled transcript and open reading frame for *Nematostella ARNT*.
(0.03 MB DOC)

Table S1 Primer sequences for amplifying pieces of *Nematostella vectensis* genes for cloning and qPCR.
(0.01 MB DOCX)

Table S2 Primer sequences for amplifying full length transcripts of *Nematostella vectensis* genes for *in vitro* transcription and translation. The “cacc” at the amino terminus of the forward primers facilitates directional cloning and is not part of the endogenous sequence.
(0.01 MB DOCX)
