# Supplementary figures and images for: Light Entrained Rhythmic Gene Expression in the Sea Anemone Nematostella vectensis: The Evolution of the Animal Circadian Clock
Source: PLoS One. 2010 Sep 21;5(9):e12805. doi: 10.1371/journal.pone.0012805 (PMC2943474; doi:10.1371/journal.pone.0012805)

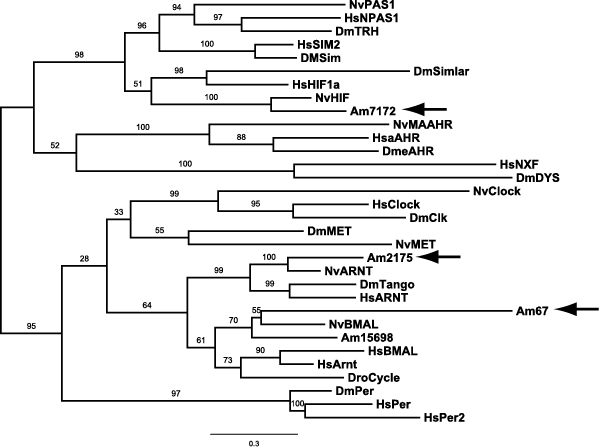

Supplement: Figure S1 — Identity of genes determined by phylogenetics previously annotated as cnidarian Period representatives by Vize [52]. In this earlier study, top BLAST matches of cnidarian gene models and ESTs to mammalian and insect Period were reported. In our analysis, we show that none of these (indicated by arrowheads in figure) are orthologs to Period genes from mammals or insects, which form a strongly supported clade (represented by DmPer, HsPer, and HsPer2). The three Acropora genes (Am) group with genes from Nematostella (Nv) that nest within three separate bHLH-PAS families: HIF, ARNT, and Bmal. Phylogenetic methods are identical to those used throughout this study. Support values above nodes indicated percentage of 1000 bootstraps. (0.04 MB DOCX) [file pone.0012805.s001.docx]

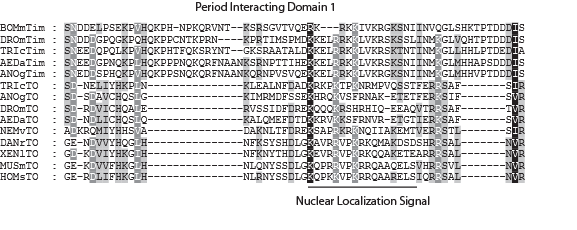

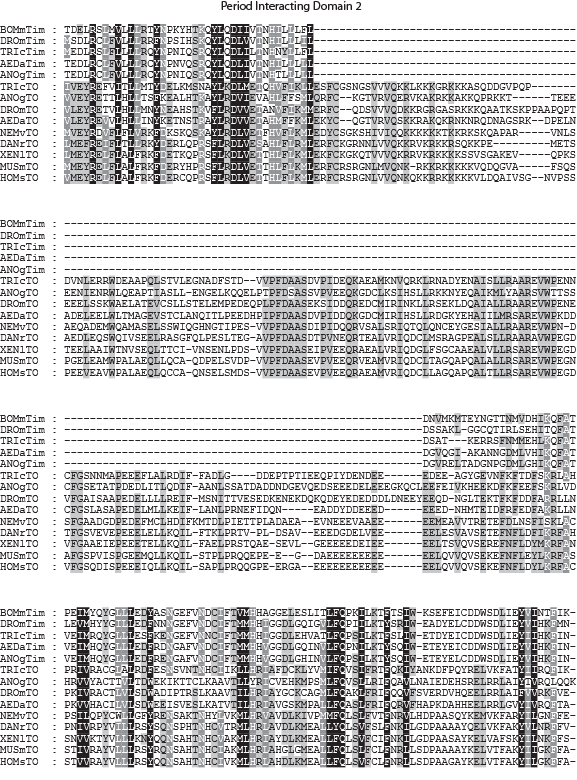


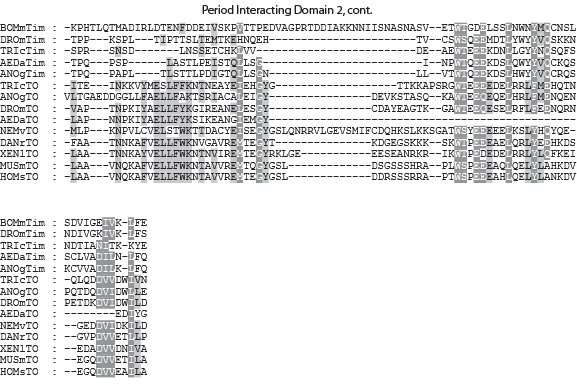


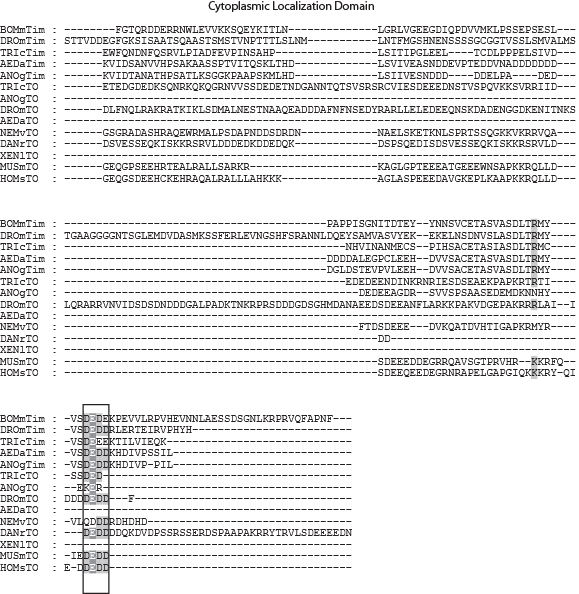

Supplement: Figure S2 — Alignment of Timeless-Timeout genes from vertebrates, insects, and N. vectensis. We have focused on three functional domains: Period interacting domain 1 that contains the nuclear localization signal, Period interacting domain 2, and the cytoplasmic interaction domain [defined in 53]. The single N. vectensis gene with similarity to the Timeless-Timeout family has considerably higher conservation with vertebrate Timeout and insect Timeout for two of the three domains than insect Timeless. As a representative example, the percent conservation of NvTimeout is higher when compared with human Timeout and Drosophila Timeout than with Drosophila Timeless (Period domain 1∶28, 32, 7%, respectively; Period Domain 2∶42, 29, 12%, respectively). The cytoplasmic localization domain could not be aligned with confidence outside of the DEDD region (which N. vectensis lacks) for these taxa and thus percent similarities could not be calculated. (0.29 MB DOCX) [file pone.0012805.s002.docx]

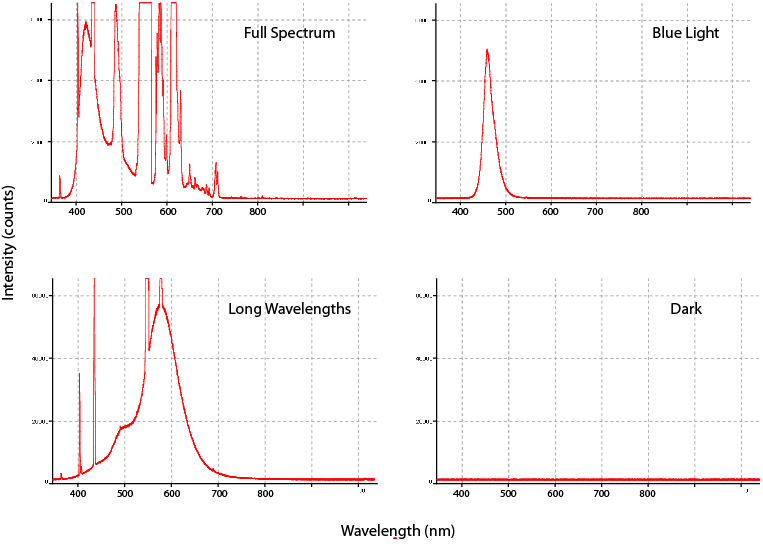

Supplement: Figure S3 — Light spectra from the four experimental treatments used in this study. Spectra were determined with a USB4000 spectrometer (Ocean Optics, Dunedin, FL). (0.06 MB DOCX) [file pone.0012805.s003.docx]
