# Supplementary material for: Light Entrained Rhythmic Gene Expression in the Sea Anemone Nematostella vectensis: The Evolution of the Animal Circadian Clock
Source: PLoS One. 2010 Sep 21;5(9):e12805. doi: 10.1371/journal.pone.0012805 (PMC2943474; doi:10.1371/journal.pone.0012805)
Supplement: Figure S4 — Assembled transcript and open reading frame for Nematostella Clock. (0.03 MB DOC) [file pone.0012805.s004.doc]

2 acagatccggaggcagaataataaagacaagcaaatgtagctagcgaaaaactggaagac

T D P E A E * * R Q A N V A S E K L E D

62 aacgacaaaatggaagcggacgactgcattgaaagcatggacgataaacacgggccgaag

N D K M E A D D C I E S M D D K H G P K

122 atatccgaaagtaaacgagttaatcggaatatgaatgaaaagaaacggcgggatagattc

I S E S K R V N R N M N E K K R R D R F

182 aacgtacttatcggagaactggcgtctatcatttcgccatcctcgcgcaaggttgataag

N V L I G E L A S I I S P S S R K V D K

242 tccacagtactgaagaaagcgattgcttgcctgaaaagccagaaggacttgtcgcccgcg

S T V L K K A I A C L K S Q K D L S P A

302 agctgttcaaagccaagggaaggttggcagccaccgtttgtgagtgatcctgagctgtgc

S C S K P R E G W Q P P F V S D P E L C

362 caactaatcatcgaagcaatggatggttttattatgagtatcgacagttcagggtctatt

Q L I I E A M D G F I M S I D S S G S I

422 agttttgtctctgataacattacatctcagttaggctatttgccagagaaaattataaac

S F V S D N I T S Q L G Y L P E K I I N

482 actcgattgagcgagtacttagagtctagggattgcgaggcgatggatgtccgactgcag

T R L S E Y L E S R D C E A M D V R L Q

542 agattatacgaacacgaacttgtgatgtatcggctccccgagtctagcgccacagccacc

R L Y E H E L V M Y R L P E S S A T A T

602 ccactgagtgacctctttgatttcagcttgtgcatgtgttatggtcctctcgtggactct

P L S D L F D F S L C M C Y G P L V D S

662 actgggttcgccgcgatgcggtgtatagcgcaggcgtatgtcatgcaaggcgtaagtcac

T G F A A M R C I A Q A Y V M Q G V S H

722 gatatgaaaacaaaacctgaaataacccggaagctgaatttggtcacactgtgttgccta

D M K T K P E I T R K L N L V T L C C L

782 gagactgagcgtccaaccaagttagtgtcttccccaagcttaaaaaaaatggaattcacc

E T E R P T K L V S S P S L K K M E F T

842 gcacgtttaactttgaactggaaattcactcatttagaccagcgtggactgtctgtgata

A R L T L N W K F T H L D Q R G L S V I

902 ggttacatgtcaaatgagctggttggttcctcgctgtatcagaacatccatccgaatgac

G Y M S N E L V G S S L Y Q N I H P N D

962 ttagagaatattacacgctatcacaagatattagtttataaaggtcgggtgaatacttgt

L E N I T R Y H K I L V Y K G R V N T C

1022 tattaccgatttctgaccaaggggcaagcctatctctggataaggagctgttgttacatc

Y Y R F L T K G Q A Y L W I R S C C Y I

1082 tcatataatcaatggaactccagacctgagttcattattgccacctccacgacggccagc

S Y N Q W N S R P E F I I A T S T T A S

1142 caagcagaagttactgctaaccaagccagaactttacaacaagacctgcagagctttgag

Q A E V T A N Q A R T L Q Q D L Q S F E

1202 aatctagaacagaagcaacttatttctaaaacttctggagtcagtagtccttctggccca

N L E Q K Q L I S K T S G V S S P S G P

1262 acagatatgccctcaaggtcctctggggaaagcacatttagtgatttgcccatgtcagat

T D M P S R S S G E S T F S D L P M S D

1322 gctgtatcagtagggagtgaaccccaaaactcaaaaccacttagtccagggagcattata

A V S V G S E P Q N S K P L S P G S I I

1382 tcagagccaagctcgatcggagaacttgttaagtcccctgccttaggaagtggtaatgca

S E P S S I G E L V K S P A L G S G N A

1442 tttaaagctagtaggctaatgtcatctggggcctctgtatcccaagtcttggattccttg

F K A S R L M S S G A S V S Q V L D S L

1502 ttgccaagtgatgtttctgatgctggttttgggtcagatgaggatcttccaaaagaaaaa

L P S D V S D A G F G S D E D L P K E K

1562 cctgtgtcaaaggcagagtcaccatggaacctccccttgccaaaagggctctcaccgaca

P V S K A E S P W N L P L P K G L S P T

1622 cagtacaaactacatgaacagctcaaggataagcatgttttactagaggagtccataaaa

Q Y K L H E Q L K D K H V L L E E S I K

1682 cgccaaattcatgagctcaataagataaagaagcaaatcgaggtaaataaggacttatgg

R Q I H E L N K I K K Q I E V N K D L W

1742 gagttcaatgtgcagattcagaagctcagggggcctggatcgaatctggagatggaagat

E F N V Q I Q K L R G P G S N L E M E D

1802 aagaatggctcaagaaaccctgataacgaaaaccttcactctgatctcagtaacctgaat

K N G S R N P D N E N L H S D L S N L N

1862 ctactccaaggagacaagcagctaaggcacttaatgactgatctcacagatgatttctcc

L L Q G D K Q L R H L M T D L T D D F S

1922 aataatggacagatgtcacttggaaaggactttctctctatgcctaccccaaaccctgct

N N G Q M S L G K D F L S M P T P N P A

1982 tacatcgatgatgagttggaagacatgattgtctgcagtagtcagtccttgactgatgac

Y I D D E L E D M I V C S S Q S L T D D

2042 tcaggggtgttattccaatttccattcagttcatccagaaattaagcatctttcaaaact

S G V L F Q F P F S S S R N * A S F K T

2102 actcttgcttattgcagggttctacttgcatttttagccttatccgagattctatttatt

T L A Y C R V L L A F L A L S E I L F I

2162 aattttacagtatttcattaataaaatttcttaaaaaaatgaactgaatttcacaagaaa

N F T V F H * * N F L K K * T E F H K K

2222 taaactagttattagcatataagtaatggaacaatagatgctcacaaatgttgggctttt

* T S Y * H I S N G T I D A H K C W A F

2282 aagttttctttattaaaaacttccttgcccaagtttaggcaatgttaggcttagccctac

K F S L L K T S L P K F R Q C * A * P Y

2342 atgctcttatgcaaaaatagagaaaatggaggtggaatattttctacaaatgaatccaaa

M L L C K N R E N G G G I F S T N E S K

2402 tctcacaaatgttttgccatgtgactctaaggcacacggcagagt 2448

S H K C F A M * L * G T R Q S
