# Supplementary material for: Light Entrained Rhythmic Gene Expression in the Sea Anemone Nematostella vectensis: The Evolution of the Animal Circadian Clock
Source: PLoS One. 2010 Sep 21;5(9):e12805. doi: 10.1371/journal.pone.0012805 (PMC2943474; doi:10.1371/journal.pone.0012805)
Supplement: Figure S5 — Assembled transcript and open reading frame for Nematostella Cycle. (0.03 MB DOC) [file pone.0012805.s005.doc]

2 ttcgttcaaccatggcttaaaaagcgatactgcccttgtttattggtactcaagttaaac

F V Q P W L K K R Y C P C L L V L K L N

62 aggtgaaccgtaattttcttcgatacgaagaagacgtgacctacgttcgcgatcgcgcca

R * T V I F F D T K K T * P T F A I A P

122 tcgaatcgaaaagacaactcgagcggaccctgagcgaaccaaacgacgatggacatgaag

S N R K D N S S G P * A N Q T T M D M K

182 cggaagtttaacgagtctattcacgatgatgatagcgattgtgatcagtcagaagtcgga

R K F N E S I H D D D S D C D Q S E V G

242 gatgccgagtctctcagagattctggtgatagcaaaggattccagaaaggagccataaag

D A E S L R D S G D S K G F Q K G A I K

302 cagaaccatagtgaaattgagaaaaggcgccgtgataagatgaacacttacatcaatgag

Q N H S E I E K R R R D K M N T Y I N E

362 ctatctacaatgatcccaatgtgtaatgcaatgtctcgcaagctggataagttaacagtt

L S T M I P M C N A M S R K L D K L T V

422 ctgagaatggctgtgcaacatatgagggctctgcgaggtagagccgtcccttttacggag

L R M A V Q H M R A L R G R A V P F T E

482 accaattacaagccagccttcttatctgatgaggatctaaaaaaccttgtgcttgaagca

T N Y K P A F L S D E D L K N L V L E A

542 gctgatggcttcttgttcgtagttggatgtgacagggggcgtatactctatgtgtcagat

A D G F L F V V G C D R G R I L Y V S D

602 tctatacaaaactcactctatctgtcacagcttgatttagtgggaaacagtttttatgat

S I Q N S L Y L S Q L D L V G N S F Y D

662 caagtgcatcctagagatgtggcaaccatcaaggaccagctgtcatcccttgaggggacc

Q V H P R D V A T I K D Q L S S L E G T

722 cccaaggagagacaagcccaggaagctgctaaaaacatcaacatctctccaaaaatagag

P K E R Q A Q E A A K N I N I S P K I E

782 acgaacaaaaatcaggggcagatgtgttctggagcacgaagatcgttcttttgccgtatg

T N K N Q G Q M C S G A R R S F F C R M

842 aagtgtggggtaaagatcaagaaaagtaaagcggacgattcagactcgacacctgaacca

K C G V K I K K S K A D D S D S T P E P

902 tgcattatgaaccgcaagagcaaggccaagcaagttggtaactccgataagaagcagttc

C I M N R K S K A K Q V G N S D K K Q F

962 tcgattgttcatttcaccggttacctaaagtcttggccgccgactggtggctctgaggac

S I V H F T G Y L K S W P P T G G S E D

1022 gaagaggacgaagacaacgacgcgcgaaaccttagctgtctcgtggccgtcggaaggctc

E E D E D N D A R N L S C L V A V G R L

1082 gtcgaggtttccgatgagatgacggacttttcccgaccggatgtcgccaagcagttcacc

V E V S D E M T D F S R P D V A K Q F T

1142 tcgcgacattcaagcgacggaaagtttatctacgtggaccagaggattgtgtcaatatgc

S R H S S D G K F I Y V D Q R I V S I C

1202 ggctacttgcctcaagaagttattggtacgtcagggtatgactacttccacccggaggac

G Y L P Q E V I G T S G Y D Y F H P E D

1262 ctggagatcgtggcacagtcacataagagcgcgctacagggagagaccgccgtatcctcc

L E I V A Q S H K S A L Q G E T A V S S

1322 tacaggttcctctgtaaatcaggtcactatattcctctgagaacaaggtcaactctgttc

Y R F L C K S G H Y I P L R T R S T L F

1382 cgtaatccatggacgaaagagatcgagtttcttgtatgcaccaacgatgtcctcactgag

R N P W T K E I E F L V C T N D V L T E

1442 tttgacttgccgccgcaaagcaacaccgtggtagtacctccaacaagccagaggccgatc

F D L P P Q S N T V V V P P T S Q R P I

1502 cttccagccccgagtccacagacactgcagaccatgcagaattcgaaaccgggagtcaag

L P A P S P Q T L Q T M Q N S K P G V K

1562 cagctattggaggtgcttcagaggcagcagggtaagggtgggaagaaaggacgagcattt

Q L L E V L Q R Q Q G K G G K K G R A F

1622 cagtacccttcatcggaggatggggaggcgtccacccctaaaccatccatgattggcacc

Q Y P S S E D G E A S T P K P S M I G T

1682 cttctcgctgaagaagcacaggtgatgagcagtcgacaaaacgacccatatcggtattcc

L L A E E A Q V M S S R Q N D P Y R Y S

1742 atacacaggcaacccgtggttctcggcagttcattgccgtcctctaccactggctcggtt

I H R Q P V V L G S S L P S S T T G S V

1802 acaggaggctacagcgcgggtcctgaaagcattgggagcagtatcgacttcagtatgatg

T G G Y S A G P E S I G S S I D F S M M

1862 aaccctggtatgtcaaccatgagcgactcgagactgcctctctaccctcaaagcgacatg

N P G M S T M S D S R L P L Y P Q S D M

1922 ggatcggtgcagtcggcaaagttttcccgcggttcgtacgggatgccggaaatgacacaa

G S V Q S A K F S R G S Y G M P E M T Q

1982 cctcacttgcttgagcaggtgattagtatgcagcatgcaatcgagtctatggaaagtccc

P H L L E Q V I S M Q H A I E S M E S P

2042 atgcagcaaagcgaggagagcatggcggttttcatgaacattctagaagaagatgccggt

M Q Q S E E S M A V F M N I L E E D A G

2102 cttggaggggaattcaatgacttgaccttcaaaagtagtaataactaaacttttaaggcc

L G G E F N D L T F K S S N N * T F K A

2162 tgactaaatatggatcgctgtagcaaatgttttctaacgattctattttagctagcgcta

* L N M D R C S K C F L T I L F * L A L

2222 tattaagggaaattgcaaaaacgtttgtagccggcgacatgagaatgttccttgactatt

Y * G K L Q K R L * P A T * E C S L T I

2282 agagactaaaaacttttgtatctcagctactggatctagcgtgcgctatgaagttttctc

R D * K L L Y L S Y W I * R A L * S F L

2342 cggctggctgaactagaaaacatctcctaccatatatcatgtatcctaatttagccaggc

R L A E L E N I S Y H I S C I L I * P G

2402 ctttactggaatcctttttctgtacatacatgacgcttttttcccgagatatagcattcg

L Y W N P F S V H T * R F F P E I * H S

2462 tgaagactattaaagcgttcgcgaaac 2490

* R L L K R S R N
