# Supplementary material for: Light Entrained Rhythmic Gene Expression in the Sea Anemone Nematostella vectensis: The Evolution of the Animal Circadian Clock
Source: PLoS One. 2010 Sep 21;5(9):e12805. doi: 10.1371/journal.pone.0012805 (PMC2943474; doi:10.1371/journal.pone.0012805)
Supplement: Figure S6 — Assembled transcript and open reading frame for Nematostella ARNT. (0.03 MB DOC) [file pone.0012805.s006.doc]

2 cgttacctcgaaaaaatcaattcatccctactctacatgactctaatcaagcaatttaaa

R Y L E K I N S S L L Y M T L I K Q F K

62 atttacaactttccaagaggtcttccaagggatttttatgctgtctaactggttgtaaat

I Y N F P R G L P R D F Y A V * L V V N

122 tagaagtatttgtaaagacgcgttgaacgtaataaggtcgatgtgtgttgatgacaacga

* K Y L * R R V E R N K V D V C * * Q R

182 ggagtatagcggccatcgatagactacgtcccttcctgagttttcctttggcgccgcaaa

G V * R P S I D Y V P S * V F L W R R K

242 ggccttgatttcacgctggatcccacacaaggcttcatgtttactcccggctctatggga

G L D F T L D P T Q G F M F T P G S M G

302 gaggagcaagatgactacgaagacgaaatgtccgaaaaatcttcgcatggtcgtctgcgg

E E Q D D Y E D E M S E K S S H G R L R

362 aaaagaaaatctaacatacaaatgatgataaaagacgaagaatctgacgatgatgacccg

K R K S N I Q M M I K D E E S D D D D P

422 gataaacatgatggcaaaataccgaggaccaacgatcccgaaaatgtcaaggacaagttt

D K H D G K I P R T N D P E N V K D K F

482 gctagggagaatcacagtgaaattgagagaagaagaagaaataagatgaatgcatacatc

A R E N H S E I E R R R R N K M N A Y I

542 aatgagttatcagacatggtcccatcatgcactggactggcaagaaaacctgataaactc

N E L S D M V P S C T G L A R K P D K L

602 acggtcctacgaatggctgtcaactacatgaagacattacgaggaacagctccccaagat

T V L R M A V N Y M K T L R G T A P Q D

662 gtgaattacaagccatcttttctatctgatcaagagctcaagcatctgatcttggaggca

V N Y K P S F L S D Q E L K H L I L E A

722 gcagatggcttcttgtttgtcgtcaactgccagactgctacagttgtttatgtctctgat

A D G F L F V V N C Q T A T V V Y V S D

782 tccatcagccctgttcttaaccaaagccagaatgcatggatgaaccagtgcttgtatgac

S I S P V L N Q S Q N A W M N Q C L Y D

842 ttgattcacccagaagacgttgagaaagtgcgagaccagctgtcatcttcaaactcgcca

L I H P E D V E K V R D Q L S S S N S P

902 gatgctggcagggtattggatatcaagactggcagtgttaagagggatgctcatggagct

D A G R V L D I K T G S V K R D A H G A

962 ctatcaagaatgtactctagtacaaggagaaacttcatctgccgtatgagacgtggtaat

L S R M Y S S T R R N F I C R M R R G N

1022 gaagtttgtgaaactcaggtcaaagaggaacatccaaaggtccctgtacttgaggatgag

E V C E T Q V K E E H P K V P V L E D E

1082 tatgctgttgtccactgcacaggatatctcaagaattggagtggcagtagtagcagtaat

Y A V V H C T G Y L K N W S G S S S S N

1142 ggtaactcaagcttccagggagaggatcctaacggtacgtcactggtcactattggcaga

G N S S F Q G E D P N G T S L V T I G R

1202 ttacagcctgctagcgtaccccacagtaacgacctcgcagaaacacctactgtaacagag

L Q P A S V P H S N D L A E T P T V T E

1262 ttcatctcaagacatagcttggatggaaagttcacatttgttgatcaaagagtcactgaa

F I S R H S L D G K F T F V D Q R V T E

1322 gtccttggctaccagccccgcgacatgctcggccagttgtgctatgacttcttccatcct

V L G Y Q P R D M L G Q L C Y D F F H P

1382 gatgacctcgaacacatgatggagagctatgaccaagtaatgaagctgaaaggccagact

D D L E H M M E S Y D Q V M K L K G Q T

1442 ctgtctgtgcgttatcgattccgctccaagactggtgactgggtgtggctaagaacaagc

L S V R Y R F R S K T G D W V W L R T S

1502 tgcttcagtttccagaacccttacacagatgaagcggagtacatagtatgcaacaacaac

C F S F Q N P Y T D E A E Y I V C N N N

1562 ctggttaacaatgattaccagcagcagatgcagccaaaccttccccagatcatggccagt

L V N N D Y Q Q Q M Q P N L P Q I M A S

1622 ccgtccggcctgccgccgccccatgctagcatgggaccgatcagctctggaagcagtagt

P S G L P P P H A S M G P I S S G S S S

1682 tcagagtactcacagatctcgccagcaggaggcatgaccagccctgagatgcaacaacaa

S E Y S Q I S P A G G M T S P E M Q Q Q

1742 tacatgcaaatgcagcaacaacaacagcagggtgtcaagcaaggtgtaggcagcgacgag

Y M Q M Q Q Q Q Q Q G V K Q G V G S D E

1802 gccatattccgtttttctgggggccccaaaggtcaacaacaaattccagaaagtggccta

A I F R F S G G P K G Q Q Q I P E S G L

1862 gatgcattggccaaggctggggagttaacagaaaagagaggcacccctgttacttcatct

D A L A K A G E L T E K R G T P V T S S

1922 acaacttcaagaatggagggacagatgaaccagcaagatttagataagacagacttctca

T T S R M E G Q M N Q Q D L D K T D F S

1982 gctggacaagcaggcagtcttctcgctgctttggttcagagaaggaatgcaatggctgca

A G Q A G S L L A A L V Q R R N A M A A

2042 gtctctaacccaggtggtccaactacaacacagtcttcgctgtacagtcagttaatatct

V S N P G G P T T T Q S S L Y S Q L I S

2102 caagtaaatggtggtatgggtatgcctcgaccaggaatgacacacccgatggggcagccg

Q V N G G M G M P R P G M T H P M G Q P

2162 acagccatgggggacctttgtccccagggaaatatggattataggaaggctgcccccgat

T A M G D L C P Q G N M D Y R K A A P D

2222 ggacgggatatgcagcaaatgggacaaaaggggaacttttctcagatgctatctcaggga

G R D M Q Q M G Q K G N F S Q M L S Q G

2282 aggggtatgccaccaggggcatggccaggaatgattgggtcagctggcactgggggcgca

R G M P P G A W P G M I G S A G T G G A

2342 gagatgccaggaatgatgcctggggccggtggtattggacagggccagcaaatgtctgga

E M P G M M P G A G G I G Q G Q Q M S G

2402 atgtacggccaagttagtgcgccgaacacctcaagtgggcgtggtgccacagggtcatac

M Y G Q V S A P N T S S G R G A T G S Y

2462 ccatattatcagtagaagtctgcatggttcatggtgctggcgatgaacatgataacatca

P Y Y Q * K S A W F M V L A M N M I T S

2522 tatagtcatagcgactagcttcctgctgctgttattgttatgatagtcgcacctgcttgc

Y S H S D * L P A A V I V M I V A P A C

2582 cactactttctcatcatctgagaagtagttatttccgaaactctaaccaaatgctttact

H Y F L I I * E V V I S E T L T K C F T

2642 tgcgaggttcacatcctaacaaacaatgtctcagtagcgaatgtatatttttgtggtaca

C E V H I L T N N V S V A N V Y F C G T

2702 tagaaaacttccctgatattctgtttctctggatcagagggggggttgccttctaaataa

* K T S L I F C F S G S E G G L P S K *

2762 tataactcgaaaattgtttttaaagcaattagacatggcagtgattggtgtaattatgaa

Y N S K I V F K A I R H G S D W C N Y E

2822 ttttaaattttgtaatggagaaaaataaattttgactgtgttcttaaagctgagacaagc

F * I L * W R K I N F D C V L K A E T S

2882 cagcagctggtgataaaaagctaaaggctgatattcttgcgaaatataagaactactgat

Q Q L V I K S * R L I F L R N I R T T D

2942 ctgtaagaa 2950

L * E
