# Supplementary material for: Light Entrained Rhythmic Gene Expression in the Sea Anemone Nematostella vectensis: The Evolution of the Animal Circadian Clock
Source: PLoS One. 2010 Sep 21;5(9):e12805. doi: 10.1371/journal.pone.0012805 (PMC2943474; doi:10.1371/journal.pone.0012805)
Supplement: Table S1 — Primer sequences for amplifying pieces of Nematostella vectensis genes for cloning and qPCR. (0.01 MB DOCX) [file pone.0012805.s007.docx]

| Gene | Cloned Fragment Primers (5’ – 3’) | qPCR Primers (5’ – 3’) |
| --- | --- | --- |
| *NvClock* | CTGTTCAAAGCCAAGGGAAG  TTGGGGTTCACTCCCTACTG | TAACCCGGAAGCTGAATTTG  GCTTGGGGAAGACACTAACTTG |
| *NvCycle* | TTCTGAGAATGGCTGTGCAA  GGGAAAAGTCCGTCATCTCA | AGGAGAGACAAGCCCAGGAAG  ACATCTGCCCCTGATTTTTG |
| *NvTimeout* | AGGCGGAAAAGGTAAGAGAAC  TTGCGGACCAAGAAAAGG | GCATTACTCCTTAGTGTCTCATCG  CCAGTAACTTGGGGAAGAAGG |
| *NvCry2* | CATGGGGGTGAGACAGAAG  TCTCTGGCATTTGAAACTGTG | GCATCTGATTTGCAGAAATGG  CTACACGGGCGAGATAGTGG |
| *NvCry1a* | CAGCAGTTAAAAACGCCAAAG  TCAGGAAACACCCAATGGAC | AAAACGCTTGGAAACTGTGG  TGGGAAAGGGTGCATAACTC |
| *NvCry1b* | GCACTGGTTCAGGAAAGACC  ACAGCCACGAGTCAGGAAAC | CAAGCTGTACGTCGTTGAGGAG  GAGGTTTGCGGACCTGTAAC |
| *NvHSC71* | CAAACGACCAAGGAAATCGT  GGACTTGTTCAGCTCCTTGC | TCGATGATCCTGGGGTAAAG  CCTGCCTCGTTCACTACCTC |
