# Supplementary material for: Light Entrained Rhythmic Gene Expression in the Sea Anemone Nematostella vectensis: The Evolution of the Animal Circadian Clock
Source: PLoS One. 2010 Sep 21;5(9):e12805. doi: 10.1371/journal.pone.0012805 (PMC2943474; doi:10.1371/journal.pone.0012805)
Supplement: Table S2 — Primer sequences for amplifying full length transcripts of Nematostella vectensis genes for in vitro transcription and translation. The “cacc” at the amino terminus of the forward primers facilitates directional cloning and is not part of the endogenous sequence. (0.01 MB DOCX) [file pone.0012805.s008.docx]

| Gene | Primers (5’ – 3’) |
| --- | --- |
| *NvClock* | caccATGGAAGCGGACGACTG  GAACCCTGCAATAAGCAAGAG |
| *NvCycle* | caccATGGACATGAAGCGGAAGTTTAAC  GTTATTACTACTTTTGAAGGTCAAGTCATTG |
| *NvARNT* | caccATGTTTACTCCCGGCTCTATGGG  CTGATAATATGGGTATGACCCTGTGG |
